# Psychological status of medical staff dedicated to nucleic acid collection in COVID-19 epidemic during closed-loop management: A cross-sectional study

**DOI:** 10.3389/fpubh.2023.1131971

**Published:** 2023-03-10

**Authors:** Mingzhu Sun, Xiaowei Li, Jie Yao, Xi Huang, Yujuan Kang, Zixuan Li

**Affiliations:** ^1^School of Nursing, Shaanxi University of Chinese Medicine, Xianyang, Shaanxi, China; ^2^Department of Nursing, Affiliated Hospital of Shaanxi University of Chinese Medicine, Xianyang, Shaanxi, China

**Keywords:** COVID-19, closed-loop management, RT-PCR test, nucleic acid collection, psychological status, influencing factors

## Abstract

**Background:**

To investigate the depression, anxiety and somnipathy situation occurred in the nucleic acid collection staff during the closed-loop management period of COVID-19. And try to understand the influencing factors of related psychological status.

**Methods:**

A cross-sectional study of 1,014 nucleic acid collection staff from seven Chinese hospitals was conducted. Various investigation methods were involved in the questionnaires to collect data, including 12-items self-made questionnaire survey of basic demographic information, 9-items patient health questionnaire depression scale (PHQ-9), 7-items generalized anxiety disorder scale (GAD-7) and Pittsburgh sleep quality index (PSQI). Data analysis was performed using SPSS version 26.0 and Excel software. Mann-Whitney U-test, Chi-square test, correlation analysis, mono-factor analysis and binary logistic regression were applied accordingly for further analysis.

**Results:**

The positive rate of depression, anxiety and sleep disorder of 1,014 nucleic acid collectors under closed-loop management were 33.5, 27.2, and 50.1%, respectively. Depression was significantly positively correlated with anxiety and sleep (*P* < 0.05). The scores of depression scale were positively correlated with the age and the fear for infection (*r* = 0.106, 0.218, both *P* < 0.05); The scores of anxiety scale were also positively correlated with the age and the fear for infection (*r* = 0.124, 0.225, both *P* < 0.05); The length of service, collection time and the degree of worry about infection and was positively correlated with the score of sleep scale (*r* = 0.077, 0.074, 0.195, both *P* < 0.05); Education level had a significant negative association with PHQ-9, GAD-7 and PSQI (*r* = −0.167,−0.172, both *P* < 0.05). Binary logistic regression analysis showed that age, technical title, education level, collection time, collection frequency, collection location, fear for infection and external environment were important influencing factors of depression, anxiety and sleep disorders.

**Conclusion:**

The results of this study suggested that when carrying out nucleic acid collection mission, managers should intervene to optimize the collection location, control the duration of each collection mission, replace the collection staff in time and pay close attention to the psychological state of the collection staff.

## 1. Introduction

A novel coronavirus disease (COVID-19), caused by Coronavirus type 2 (SARS-CoV-2) emerged in Wuhan, China in December 2019, has become the third coronavirus in the past 20 years since the outbreak of severe acute respiratory syndrome coronavirus (SARS-CoV) in 2002 and the Middle East outbreak of respiratory syndrome coronavirus (MERS-CoV) in 2012. With rapidly spread all over the world and concomitant mortality burden, the COVID-19 outbreak represents a global public health issue which unseen in the last century (1–4). So far, the world has been witnessed four waves of viruses: Alpha variant (B.1.1.7), Beta variant (B.1.351), Gamma variant (P.1) and Delta variant (B.1.617.2). However, the new strain, Omicron (B.1.1.529), was discovered for the first time in South Africa in November 2021 (5). This strain has the characteristics of fast transmission, strong infectivity, high pathogenicity and short incubation period (6). Therefore, we need more rapid and immediate responses in healthcare to avoid more infections.

In December 2021, a case of localized Omicron variant occurred in Xi'an, Shaanxi. There were 2,080 new cases of coronavirus pneumonia confirmed within 40 days, the largest local outbreak in a megacity since the Wuhan outbreak (7). According to the COVID-19 Diagnostic Guide issued by China and related research, a positive real-time reverse transcription polymerase chain reaction (RT-PCR) test is considered the main criterion for the diagnosis of COVID-19 (8, 9). Nucleic acid collectors are one of the important pillars during the pandemic and play a key role during the pandemic.

In order to prevent the further spread of the epidemic, the medical institutions actively arranged nucleic acid collection teams to carry out wide range nucleic acid sampling throughout the city and closed-loop management for nucleic acid collection employees in accordance with the local epidemic prevention and control procedures. Closed-loop management is a management method formed by integrated information system, closed-loop system, management control and management closure principle. It can be effectively used in epidemic prevention and control (10). Point-to-point management allowed for closed-loop hospital locations, closed-loop roadways, closed-loop lodging sites, closed-loop epidemics and closed-loop patient status (hospital to hotel accommodation sites, hospital to quarantine sites).

During the sampling period, due to the large population of Xi'an, the sampling workload is extremely heavy, and the maximum sample size can reach 8.92 million people per day. The nucleic acid collectors need to start the preparation work at 3 am every day. The sampling site conditions are rudimentary, most of them are mobile sampling points which are temporarily constructed outdoors. And the weather is cold during the epidemic, the protective suits are too thin to keep warm. The working hours are also uncertain, most people work more than 8 h a day. Due to strict protection requirements, medical staff can barely drink water when sampling, and it is inconvenient to go to the toilet. All these factors may cause high work pressure on both physical and mental aspects among nucleic acid collectors.

Previous studies have found that frontline workers are prone to a range of psychological problems, including fear, depression, anxiety, post-traumatic stress symptoms and insomnia during high-risk and stressful situations during the pandemic (11–13). For example, a large proportion of healthcare workers battling SARS suffer from depression, anxiety and sleep problems (14, 15). Recent studies have shown that health workers have higher psychological problems during COVID-19 than in past epidemics (16). Several studies have shown that physical, emotional, and mental health can all be impaired by overwork (17–20). The potential negative psychological impact is not only detrimental to the growth of medical staff, but also may reduce effective response to emergencies (11, 21).

At present, there have been many investigations on the anxiety and depression of medical staff in the fight against the epidemic. But during the strict closed-loop management, nucleic acid collectors face great physical and mental challenges, and there are few studies on the impact of such management methods on psychological state. The purpose of this paper was to conduct a preliminary psychological evaluation of nucleic acid collectors who participated in closed-loop management during the Omicron epidemic in Xi'an, and analyze the influencing factors to provide a scientific basis for later psychological recovery.

## 2. Method

### 2.1. Setting and sampling

This survey based on convenience sampling was conducted for medical personnel for nucleic acid collection during closed-loop management in 7 Grade III Grade A hospitals in Shaanxi Province from March 25 to April 18, 2022. To facilitate the sample collection, all personnel participated in nucleic acid collection-related training, informed consent and voluntary participation in this study. Thompson's study suggested a sample size of 10–15 times the questionnaire items for the structural equation model (22). Forty seven items made up the self-administered questionnaire, which had a total sample size of 705 participants. Due to incomplete questionnaires, the sample size was increased by 20% to a total of 846; nevertheless, the final sample size was 1,028 individuals.

### 2.2. Ethical approval

All personnel volunteered to participate in this study and were allowed to withdraw during the process. The electronic information submitted was anonymous and only researchers had access to the data. The Ethics Committee of the Affiliated Hospital of Shaanxi University of Traditional Chinese Medicine, Shaanxi Province, China approved this study (Ethical number:2022-0516).

### 2.3. Data collection

Wenjuanxing, an online crowdsourcing platform on the Chinese mainland, was used to make the questionnaire because of the outbreak. It was then sent to the WeChat group, which is a public social networking tool in China, for the staff who collect nucleic acids. The WeChat group was informed of the objective and importance of this survey and one response is allowed from a given IP address. After the questionnaire had been reviewed and examined by two quality controllers, the entire survey results were collected from the questionnaire star platform. Those who chose the same option in questionnaires and those who missed >10% of items were excluded. After filtering, 1,014 questionnaires were included in the subsequent analysis with a response rate of 98.64%.

### 2.4. Measurements

The survey consisted of the following four parts: Sociodemographic data, Patient health questionnaire-9 (PHQ-9), Generalized anxiety disorder 7-item (GAD-7) Scale and Pittsburgh sleep quality index (PSQI).

#### 2.4.1. Demographic information of survey respondents

A self-administered general data questionnaire was used, mainly including gender, age, occupation, years of work, education level, technical title, nucleic acid collection attendance frequency in the past 3 months (hereinafter referred to as “collection frequency”), continuous collection time per attendance (hereinafter referred to as “collection time”), a regular presence at the site of nucleic acid collection (hereinafter referred to as “collection place”), and whether external environmental factors (winter, summer, rain, wind) affect nucleic acid collection (hereinafter referred to as “external environment”). The degree of concern about infection is hereinafter referred to as “the degree of concern.”

#### 2.4.2. Patient health questionnaire-9 (PHQ-9)

The Chinese Patient Health Questionnaire-9 (PHQ-9), a self-measuring reporting method used to assess the severity of depression, is widely used as an open screening tool for depression in different healthcare and community environments (23). PHQ-9 is composed of 9 items, with a Likert score of 4, divided into scores of 0 (none at all), 1 (a few days), 2 (most of the time), and 3 (almost every day), for a total score of 27 points. The higher the score, the more severe the depression. Scoring criteria: no depression (0~4), mild depression (5~9), moderate depression (10~14), severe depression (15~27). The psychometric characteristics of the PHQ-9 are reliable to measure depression allied with clinical features with strong consistency (Cronbach's alpha = 0.86) (24). The study concluded that a score of 10 was the optimal threshold for PHQ-9 with a sensitivity of 92.8% and specificity of 95.7% (25). Therefore, a score of 10 on the PHQ-9 questionnaire indicated the presence of depression.

#### 2.4.3. Generalized anxiety disorder 7-item (GAD-7) scale

The generalized anxiety scale, developed by Spitzer et al. (26) in 2006 to identify anxiety disorders with optimal reliability and validity, assesses the severity of anxiety symptoms over 2 weeks. GAD-7 is composed of seven items, with a Likert score of 4, divided into scores of 0 (none at all), 1 (a few days), 2 (most of the time), and 3 (almost every day), for a total score of 21 points. The higher the score, the more severe the anxiety suppression. Scoring criteria were no anxiety (0~4), mild anxiety (5~9), moderate anxiety (10~14), and severe anxiety (15~21). On the other hand, the psychometric characteristics of the GAD-7 are also reliable to measure anxiety allied with clinical features with strong consistency (Cronbach's alpha = 0.92) and good test-retest reliability (*r* = 0.88) (26). In this questionnaire, people with GAD-7 score ≥10 were very likely suffering anxiety, a score of 10 was considered the optimal cut-off value for the GAD-7 with a sensitivity of 89% and specificity of 82% (27).

#### 2.4.4. Pittsburgh sleep quality index, PSQI

This scale was compiled by Buysse et al. (28) and translated into Chinese (29) to evaluate the sleep quality. The scale can evaluate the sleep situation of nearly a month and contains 18 items and 7 dimensions, including sleep quality, time to sleep, sleep time, sleep efficiency, sleep disorder, hypnotics and daytime dysfunction. The responses to the items are weighted on a scoring scale between 0 and 3. The total PSQI score is from 0 to 21 points. The seven component scores are then summed to yield a global PSQI score, which has a range of 0–21, higher scores indicate worse sleep quality. The scoring criteria were normal sleep quality (0~5), mild sleep disorder (6~10), moderate sleep disorder (11~15), and severe sleep disorder (16~21). The PSQI has internal uniformity and a reliability coefficient (Cronbach's alpha) of 0.90 for its seven components, the overall PSQI global score correlation coefficient for test-retest reliability was 0.87 (30). When PSQI is applied in different populations, in order to achieve better screening effect, it is necessary to adjust the judgment cut-off. Applying PSQI ≥ 7 as a reference value for sleep quality problems in Chinese adults (31, 32).

### 2.5. Data analysis

The collected data were analyzed using SPSS version 26 and Excel software. The distribution of PHQ-9, GAD-7, and PSQI scores in the general population did not adhere to a normal distribution, according to a first evaluation of the measured data's normality. The median and quartile were used to represent *M* (*P*_25_, *P*_75_), and the non-parametric rank sum test (Mann Witney U) was employed for intergroup comparison, and count data were expressed as ratio or composition ratio (%). Second, the differences between groups were compared using the Chi-square test (χ^2^), and all of the variables' correlations were determined using Pearson's correlation coefficients. Third, monofantor analysis and binary logistic regression were used to analyze the influencing factors of depression, anxiety and sleep disorder. The OR value and 95% *CI* were calculated, with *P*-values < 0.05 being regarded as statistically significant (2-sided tests).

## 3. Results

### 3.1. General participant characteristics

The demographic and work-related characteristics and scores of 1,014 nucleic acid collections are shown in Table 1. Demographic data analysis by gender showed that 84.3% of the 1,014 participants were female; 73.4% were nurses; 35.1% were 31–40 years old; 66.2% were undergraduates; junior professional titles accounted for the majority, 69.8%; the participants acquired once per day (36.4%); 55.4% were community-based; the acquisition time was 6–8 h per day, accounting for 47.5%; 74.5% would be influenced by the external environment; and 42.0% were generally concerned about infection.

**Table 1:** Demographic characteristics of study sample [*N* = 1,014(%)].

**Variable**	**Gender**		***N***	***χ^2^***	***P***
	**Male (%)**	**Female (%)**			
Professional	Doctor	84 (52.8)	123 (14.4)	207	187.727	< 0.001
	Nurse	48 (30.2)	696 (81.4)	744		
	Medical student	27 (17.0)	31 (3.6)	58		
	Medical technician	0	5 ( 0.6)	5		
Age (years)	≤ 25	38 (23.9)	152 (17.8)	190	4.304	0.352
	26~ < 30	46 (28.9)	295 (34.5)	341		
	31~ < 40	54 (33.9)	302 (35.3)	356		
	41~ < 50	18 (11.4)	85 (9.9)	103		
	≥50	3 (1.9)	21 (2.5)	24		
Education level	Junior college or below	50 (31.5)	201 (23.5)	251	25.463	< 0.001
	College	79 (49.8)	583 (68.2)	662		
	postgraduate or above	30 (18.7)	71 (8.3)	101		
Professional title	Basic	105 (66.0)	603 (70.5)	708	3.534	0.171
	Advance	45 (28.3)	227 (26.6)	272		
	Senior	9 (5.7)	25 (2.9)	34		
Length of service (years)	≤ 1	41 (25.8)	139 (16.3)	180	10.943	0.027
	2~5	40 (25.1)	192 (22.5)	232		
	6~10	39 (24.5)	265 (31.0)	304		
	11~15	24 (15.1)	147 (17.1)	171		
	>15	15 (9.5)	112 (13.1)	127		
Acquisition frequency (days)	One time	51 (32.1)	318 (37.3)	369	7.814	0.020
	Many times	43 (27.0)	283 (33.1)	326		
	Once/every other day	65 (40.9)	254 (29.6)	319		
Collection site	community	97 (61.0)	465 (54.4)	562	6.568	0.161
	School	19 (11.9)	113 (13.2)	132		
	Mobile Cabin Hospital	14 (8.9)	69 (8.1)	83		
	Hospital	24 (15.1)	134 (15.7)	158		
	Door-to-Door	5 (3.1)	74 (8.6)	79		
Collection time (h/d)	< 4	29 (50.0)	194 (41.8)	223	2.198	0.532
	4~ < 6	35 (22.8)	190 (27.2)	225		
	6~ < 8	83 (19.1)	399 (21.3)	482		
	>8	12 (8.1)	72 (9.7)	84		
External environment	Yes	118 (74.2)	637 (74.5)	755	0.921	0.504
	No	41 (25.8)	218 (25.5)	259		
Worry about the degree	No worry	32 (20.2)	144 (16.7)	176	3.267	0.352
	General worry	57 (35.8)	369 (43.2)	426		
	More worried	49 (30.8)	248 (29.1)	297		
	Very worried	21 (13.2)	94 (11.0)	115		

### 3.2. Score and prevalence of PHQ-9, GAD-7 and PSQI

PHQ-9, GAD-7 and PSQI of the nucleic acid collectors were statistically analyzed by sex. The results showed that the median *M* (*P*_25_, *P*_75_) score of PHQ-9 was 7 (1, 17) for males and 6 (2, 14) for females; GAD-7: 4 (0, 13) for males and 3 (0, 11) for females; PSQI was 6 (3, 8) for males and 7 (4, 9) for females. The positive rates of depression, anxiety and sleep disorders among nucleic acid collectors were 33.5, 27.2, and 50.1%, respectively; there was a significant difference between male and female groups in sleep disorder (*P* < 0.05) (Table 2).

**Table 2:** Positive rate of depression, anxiety and sleep disorders among 1,014 nucleic acid collectors under closed-loop management [*N* = 1,014(%)].

**Variables**	**Gender**	**Positive (%)**	**Degree**				***Z***	***P***
			**None (%)**	**Light (%)**	**Medium (%)**	**Heavy (%)**		
Depression	Male	340 (33.5)	66 (41.5)	36 (22.6)	7 (4.4)	50 (31.5)	−0.365	0.715
	Female		357 (41.8)	215 (25.1)	70 (8.1)	213 (24.9)		
Anxiety	Male	276 (27.2)	82 (51.6)	27 (17.0)	23 (14.5)	27 (16.7)	−0.555	0.579
	Female		476 (55.7)	153 (17.9)	116 (13.5)	110 (12.9)		
Sleep	Male	508 (50.1)	75 (47.2)	64 (40.3)	17 (10.7)	3 (1.8)	−2.555	0.011
	Female		324 (37.9)	404 (47.2)	102 (11.9)	25 (3.0)		

^*^P < 0.05, ^**^P < 0.01.

### 3.3. Correlation analysis of depression, anxiety and sleep quality

PHQ-9, GAD-7, and PSQI were found to be positively correlated (*P* < 0.05) in a correlation analysis. Age and fear for infection were positively correlated with depression and anxiety scores (*P* < 0.05). Years of work, collection time and fear for infection were positively correlated with sleep disorder scores (*P* < 0.05). Notably, education level had a significant negative association with PHQ-9, GAD-7 and PSQI (*P* < 0.05), as can be found in Table 3.

**Table 3:** Correlations for all variables.

**Variables**	**Depression**	**Anxiety**	**Sleep**	**Age**	**Length of service**	**Education level**	**Acquisition frequency**	**Collection time**	**Worry about the degree**
Depression	1.000								
Anxiety	0.885^**^	1.000							
Sleep	0.333^**^	0.289^**^	1.000						
Age	0.106^**^	0.124^**^	0.048	1.000					
Length of service	−0.027	−0.016	0.077^*^	0.788^**^	1.000				
Education level	−0.167^**^	−0.172^**^	−0.038	0.038	−0.016	1.000			
Acquisition frequency	0.046	0.050	0.016	0.004	0.018	−0.028	1.000		
Collection time	0.009	0.002	0.074^*^	−0.011	−0.024	−0.042	−0.048	1.000	
Worry about the degree	0.218^**^	0.225^**^	0.195^**^	0.03	−0.001	−0.048	0.021	−0.011	1.000

* *P* < 0.05,

** *P* < 0.01.

### 3.4. Monofactor analysis

Monofactor analysis revealed significant differences between the type of personnel, age, education level, technical title, collection time, collection frequency, collection location, external environment and worry level and the positive rate of depression and anxiety (all *P* < 0.05). There were statistically significant differences between the positive rate of sleep disorders and different education levels, collection frequency, external environment and worry level (all *P* < 0.05), as indicated in Table 4.

**Table 4:** Monofactor analysis of depression, anxiety and sleep disorder in 1,014 nucleic acid collectors under closed-loop management [*N* = 1,014(%)].

**Variables**	***N***	**Depression [*n*(%)]**	**χ^2^**	***P***	**Anxiety [*n*(%)]**	**χ^2^**	***P***	**Sleep[*n*(%)]**	**χ^2^**	***P***
**Gender**										
Male	159	57 (35.8)	0.455	0.500	50 (31.4)	1.701	0.192	69 (43.4)	3.388	0.066
Female	855	283 (33.1)			226 (26.4)			439 (51.3)		
**Professional**										
Doctor	207	77 (37.1)	11.556	0.009	68 (32.8)	13.574	0.004	100 (8.3)	4.101	0.251
Nurse	744	253 (34.0)			202 (27.2)			380 (51.1)		
Medical student	58	8 (13.8)			5 (8.6)			24 (41.4)		
Medical technician	5	2 (40.0)			1 (20.0)			4 (80.0)		
**Age (years)**										
≤ 25	190	68 (35.8)	47.402	< 0.001	62 (32.6)	72.135	< 0.001	96 (50.5)	4.529	0.339
26~ < 30	341	98 (27.3)			78 (22.9)			169 (49.6)		
31~ < 40	356	104 (29.2)			68 (19.1)			174 (48.9)		
41~ < 50	103	49 (47.6)			49 (57.6)			60 (58.2)		
>50	24	21 (87.5)			19 (79.2)			9 (37.5)		
**Education level**										
Junior college or below	251	123 (49.0)	42.818	< 0.001	106 (42.2)	38.308	< 0.001	132 (52.5)	6.125	0.047
College	662	193 (29.1)			150 (22.7)			337 (50.9)		
postgraduate or above	101	24 (23.8)			20 (19.9)			39 (38.6)		
**Professional title**										
Basic	708	264 (37.2)	17.944	< 0.001	230 (32.5)	40.780	< 0.001	351 (49.6)	0.532	0.766
Advance	272	63 (23.2)			34 (12.5)			141 (51.8)		
Senior	34	13 (38.2)			12 (35.3)			16 (47.1)		
**Length of service (years)**										
≤ 1	180	61 (33.9)	5.441	0.245	57 (31.7)	11.120	0.025	78 (43.3)	4.834	0.305
2~5	232	90 (38.8)			74 (31.9)			124 (53.4)		
6~10	304	94 (31.0)			70 (23.0)			158 (52.0)		
11~15	171	50 (29.2)			36 (21.1)			84 (49.1)		
>15	127	45 (35.4)			39 (30.7)			63 (49.6)		
**Collection time (h/d)**										
< 4	223	81 (36.3)	31.637	< 0.001	72 (32.3)	38.867	< 0.001	108 (48.4)	2.135	0.545
4~ < 6	225	77 (34.2)			63 (28.0)			106 (47.1)		
6~ < 8	482	133 (27.6)			98 (20.3)			248 (51.5)		
>8	49	43 (87.8)			43 (87.8)			46 (93.9)		
**Acquisition frequency (days)**										
One time	369	86 (23.3)	45.478	< 0.001	76 (20.6)	32.278	< 0.001	204 (55.3)	9.437	0.009
Many times	326	154 (47.2)			126 (38.7)			165 (50.6)		
Once/every other day	319	100 (31.3)			74 (23.2)			139 (42.3)		
**Collection site**										
community	562	112 (19.9)	209.404	< 0.001	67 (11.9)	287.183	< 0.001	288 (51.2)	5.199	0.267
School	132	63 (47.7)			59 (44.7)			56 (42.4)		
Mobile cabin hospital	83	76 (91.6)			75 (90.4)			46 (55.4)		
Hospital	158	76 (48.1)			68 (43.0)			82 (51.9)		
Door-to-door	79	13 (16.5)			7 (8.9)			36 (45.6)		
**External environment**										
Yes	755	277 (36.7)	13.228	< 0.001	219 (29.0)	4.768	0.029	418 (55.4)	32.783	< 0.001
No	259	63 (24.3)			57 (22.0)			90 (37.4)		
**Worry about the degree**										
No worry	176	58 (33.0)	46.452	< 0.001	53 (30.1)	53.346	< 0.001	60 (34.1)	30.483	< 0.001
General worry	426	105 (24.6)			79 (18.5)			208 (48.8)		
More worried	297	111 (37.4)			84 (28.3)			168 (56.6)
Very worried	115	66 (57.9)			60 (52.2)			72 (62.6)

### 3.5. Binary logistic regression

The following independent variables were included in the binary logistic regression model with whether depression, anxiety and sleep were abnormal as dependent variables (values of 1 for yes and 0 for no). Each variable was based on “0.” The reference group was assigned to the category of medical personnel (0 for doctors), education level (0 for junior college students), technical title (0 for junior students), collection time (0 for < 4 h/day), collection frequency (0 for once/every other day), and collection location (0 for communities), Worry level (no worry is 0).

#### 3.5.1. Overall fit of the model

The goodness-of-fit of this paper focused on the H-L index to test the goodness-of-fit of the logistic model. When the significance is >0.05, it indicates that the model fits well, and when the significance is < 0.05, it suggests that the model fits poorly, and the model test results are shown in Table 5.

**Table 5:** Model goodness of fit test.

**Variables**	**Chi square**	**Degree of freedom**	**Significance**
Depression	11.510	8	0.174
Anxiety	12.127	8	0.146
Sleep	7.202	5	0.515

#### 3.5.2. Binary logistic model results

**Figure 1:**
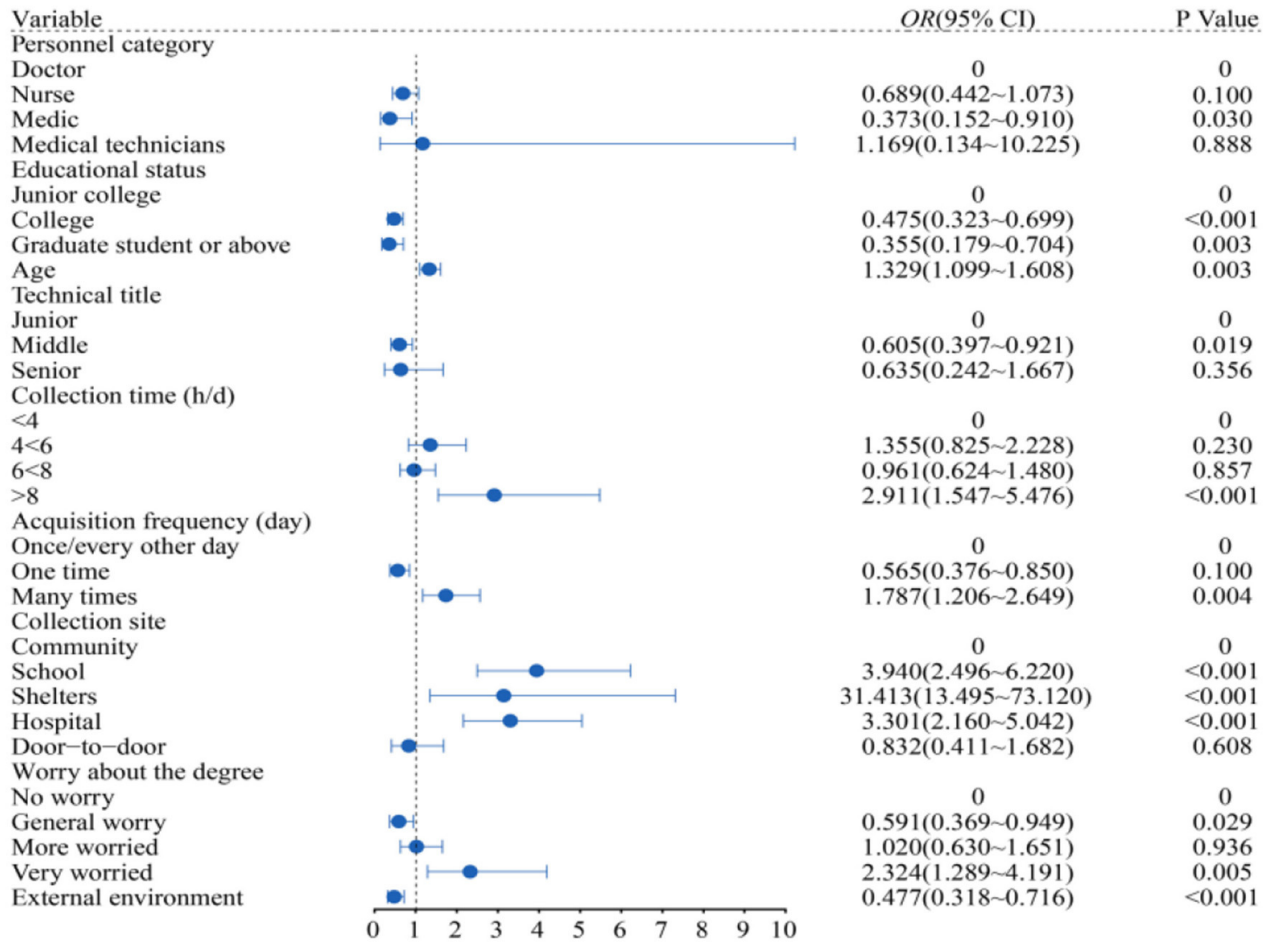
Results of binary logistic model to predict depression factors.

Figure 1 depicts the depression of medical students as being 0.373 times lower than that of doctors (95% *CI*: 0.152 to 0.910); education level is the protective factor for depression. Undergraduate and graduate students with higher education backgrounds had 0.475 times lower depression levels compared to undergraduates (95% *CI*: 0.323 to 0.699); 1.329 times higher depression prevalence per additional age unit (95% *CI*: 1.099 to 1.608); and 0.605 times lower intermediate levels compared to junior level (95% *CI*: 0.397 to 0.921); the longer the collection time, the higher the depressive mood. The risk of depressive mood of those who collected more than 8 h per day was 2.911 times higher than that of those who collected 4 h per day (95% *CI*: 1.547 to 5.476); the number of people with multiple collection times/day was 1.787 times that of people with one collection every other day (95% *CI*: 1.206 to 2.649); The people in the mobile cabin hospital were the most depressed, 31.413 times as many as those in the community (95% *CI*: 13.495 to 73.120); 2.324 times (95% *CI*: 1.289 to 4.191) of those who were very worried about infection than those who were not; the external environment could affect the depression of the collectors (95% *CI*: 0.318 to 0.716) (All *P* < 0.05).

**Figure 2:**
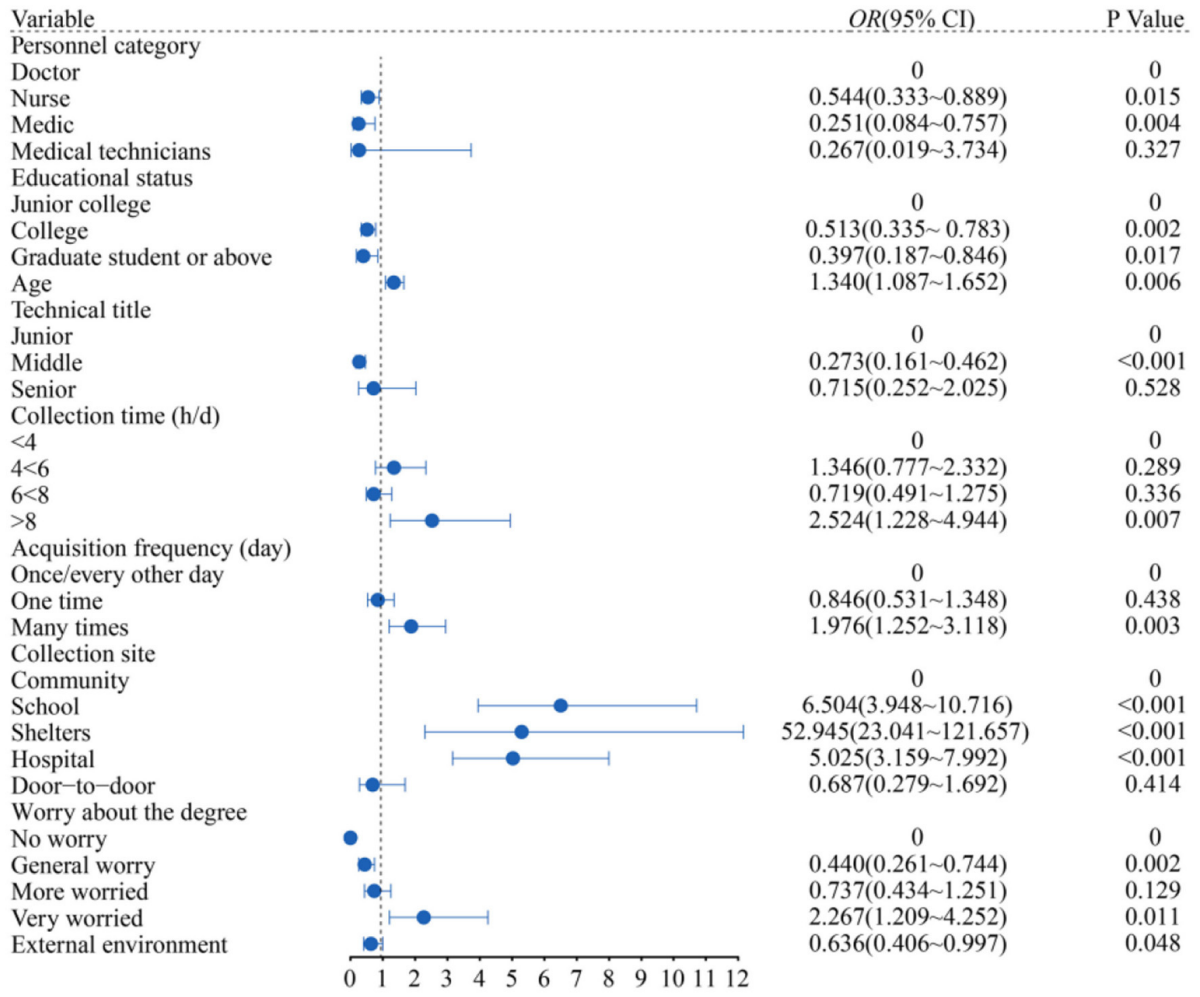
Results of binary logistic model to predict anxiety factors.

Figure 2 depicts the anxiety of nurses as being 0.544 times lower than that of doctors (95% *CI*: 0.333 to 0.889), and that of medical students was 0.251 times lower than that of doctors (95% *CI*: 0.084 to 0.757); the degree of education was a protective factor of anxiety. Compared with college students, the anxiety of undergraduates and postgraduates with higher education is 0.513 times lower (95% *CI*: 0.335 to 0.783); the incidence of anxiety increased 1.340 times (95% *CI*: 1.087 to 1.652) with the increase in age; Compared with primary professional titles, intermediate professional titles decreased by 0.237 times (95% *CI*: 0.161 to 0.462); The longer the collection time, the greater the inhibition of anxiety was. The risk of depression was 2.542 times (95% *CI*: 1.228 to 4.944) higher for those who collected more than 8 h a day than those for collected 4 h a day; the number of people with multiple collection times/day was 1.976 times that of people with one collection every other day (95% *CI*: 1.252 to 3.118); people in mobile cabin hospital were the most anxious, 52.945 times as much as those in the community (95% *CI*: 23.041 to 121.657); 2.267 times (95% *CI*: 1.209 to 4.252) of those who were very worried about infection than those who were not; the external environment affected the anxiety of the collectors (95% *CI*: 0.406 to 0.997) (All *P* < 0.05).

**Figure 3:**
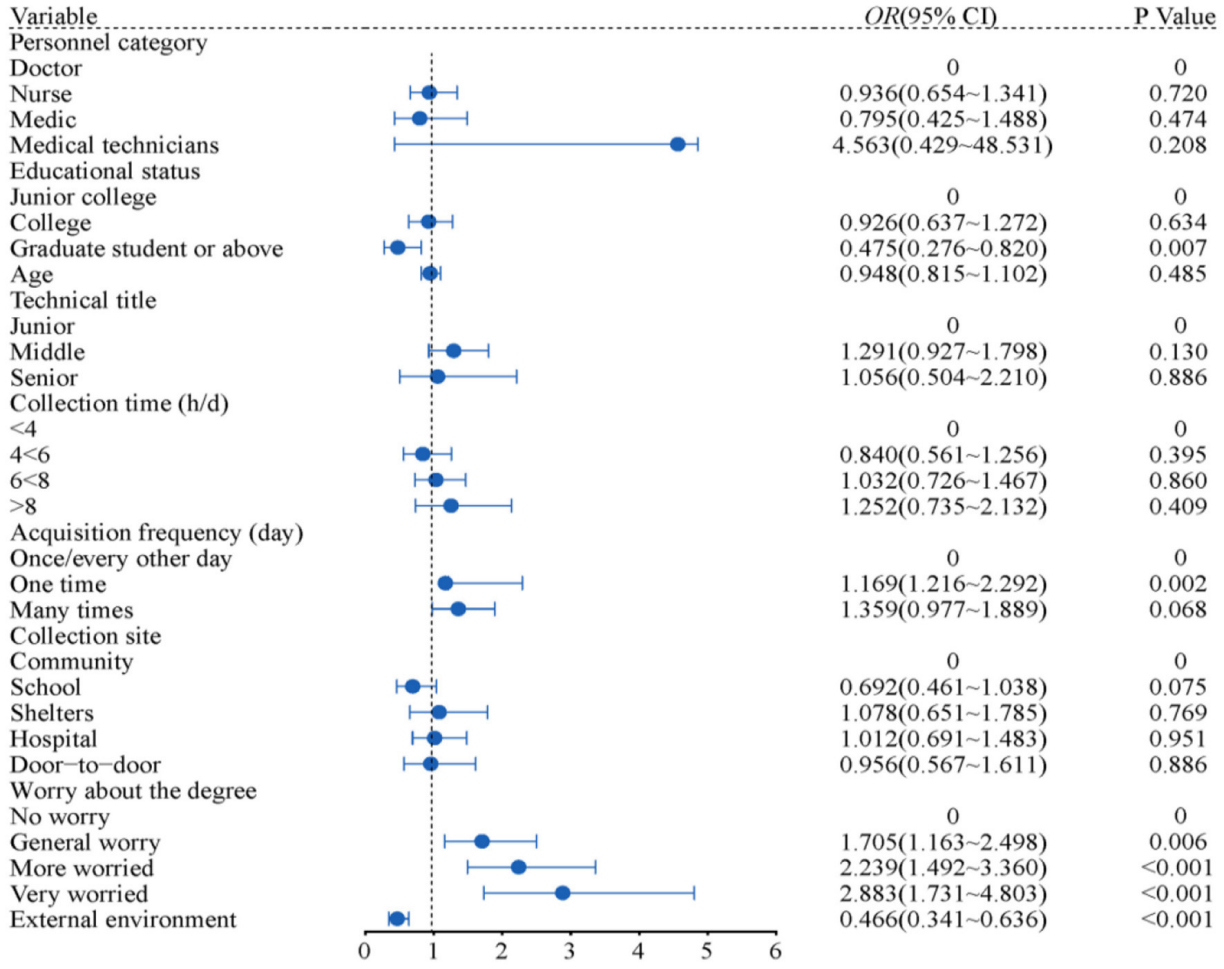
Results of binary logistic model to predict sleep disorder factors.

Figure 3 depicts that education level is the protective factor for sleep disorder. Compared with college students, the sleep disorder of postgraduates and above decreased by 0.475 times (95% *CI*: 0.276–0.820); The personnel who collected once a day were 1.169 times as many as those who collected once every other day (95% *CI*: 1.216 to 2.292); People who were very worried about infection were 2.883 times more likely to have sleep disorders than others (95% *CI*: 1.731 to 4.803); The external environment can affect the sleep of the collector (95% *CI* 0.341 to 0.636) (All *P* < 0.05).

## 4. Discussion

This study analyzed the psychological status of 1,014 nucleic acid collection staff in Shaanxi Province under closed-loop management through monofactor and binary logistic regression models. And the research found a high proportion of nucleic acid collectors with depression, anxiety and sleep disorders during closed-loop management. Age, technical title, education level, collection time, collection frequency, collection location, fear for infection and external environment also affected the psychological state. The PHQ-9, GAD-7 and PSQI used in this study are mainly used for clinical screening for depression, generalized anxiety disorders and sleep disorders. When PHQ-9 and GAD-7 scores ≥ 10 points, PSQI ≥ 7 points, usually considered that the patients may have depressed mood, anxiety or sleep disorder problems and need further diagnosis (25, 26).

First, the incidence of depression, anxiety and sleep disorders among nucleic acid collection personnel throughout the closed-loop management period were 33.5, 27.2, and 50.1%, respectively. Previous research showed that during the epidemic, 14.3% of clinical doctors and nurses suffered from depression, 11.2% from anxiety (33), and 61.67% (34) from sleep disorders. During the non-epidemic period, the incidence of depression, anxiety and sleep disorders of medical staff were 1.98%, 6.52% (35), and 39.2% (36), respectively. Moreover, compared with more research data from other countries on the incidence of depression, anxiety and sleep disorders among medical personnel during the COVID-19 pandemic. The incidence of depression, anxiety and sleep disorders were 36, 34, and 52%, respectively in Spanish medical personnel (37); 37, 23, and 34% in Latin America (38); 55, 51, and 28% in Africa (39); 34%, 46% (40), and 40% (41) in Eastern Europe. And the prevalence of depression, anxiety and sleep disorders among health-care workers in south-east Asia were generally lower than other regions during the pandemic, which were 14, 23, and 18% (42), respectively. Therefore, the prevalence of mental health symptoms during the COVID-19 pandemic is not homogeneous across different regions. Compared with the results in this study, the incidence of depression and anxiety in above countries are much higher. On the one hand, different research tools may lead to differences results. On the other hand, since the COVID-19 outbreak, the world has experienced many waves of the epidemic, medical staff's mental state is also different from the beginning. Moreover, the different incidence of depression, anxiety and sleep disorders between different countries are more likely related to region, cultural and political factors. For instance, African health-care workers have the highest rates of depression and anxiety in these countries, and it may be that in economically underdeveloped areas, the incidence of infectious diseases is high, health facilities are poor and mental health receives less attention (39). These are likely to lead to an increase in the incidence of depression and anxiety in health-care workers. On the contrary, the incidence of sleep disorders among nucleic acid collection personnel in this study is higher than in other countries. It was hypothesized that researchers and nucleic acid collectors were engaged in long-term epidemic prevention, which leading to high mental stress and heavy workload. In addition, frequent shifts, lack of sleep also increased the risk of infection during closed-loop management. The sudden outburst of this highly infectious disease and the containment measures such as quarantine and social distancing have posed immense pressure on the work and life of the health-care workers (43). This could lead to an increased incidence of sleep disorders among the nucleic acid collection staff.

Second, this study examined the correlation between the research variables among the nucleic acid collection staff. The higher the score on the depression and anxiety scale, the higher the total score of sleep disorder, indicating that medical staff sleep quality is poor. At the same time, when the sleep disorder score increased, the occurrence of depression and anxiety increased. The age of the nucleic acid collection staff was positively related to depression and anxiety, indicating that the older the patient, the more likely he or she was to suffer from depression and anxiety, the present study findings are in line with other reports (35, 44). Younger adults, however, appear to be more susceptible to sleeplessness and anxiety, according to Wang et al. (45). The discrepancy may be caused by older medical staff members' worse health state in terms of nucleic acid collection and their decreased tolerance and capacity to adjust to cold and demanding work situations compared to those under the age of 40. Depression, anxiety and sleep disorders were negatively correlated with education level, that is, the higher the education level, the lower the incidence of depression, anxiety and sleep disorders. Among people suffering from serious physical health problems, those with higher education were less likely to experience symptoms of depression and anxiety than those with lower education. Higher levels of education help to regulate the relationship between psychological and stress responses, thus reducing the occurrence of depression and anxiety (46, 47). The findings showed that fear for infection was positively associated with depression, anxiety and sleep disorders, which was because the outbreak of COVID-19 is the largest public health emergency in China in the past decade (48). At the same time, the developing Omicron variant has a high incidence and expands quickly (49). In the short term, there were many more confirmed cases in Xi'an, which can add to their psychological strain.

By analyzing data from a binary logistic regression model, we discovered greater positive rates of depression and anxiety among the mobile cabin hospital collecting staff than at other collection locations in the current research. The results of the analysis revealed that the collection personnel had extended direct contact with the sampled subjects (positive patients) and that this increased both parties' risks of infection or cross-infection compared to those in the community or schools. According to other research, medical professionals working in high-risk situations are more likely to experience depression, anxiety, and sleep difficulties as well as higher levels of “fear about infection” than those working in low-risk conditions (50). Similar to door-to-door nucleic acid collection, floor-by-floor sampling by medical professionals raises the risk of aberrant psychological situations under heavy load and external environmental effects. As evidenced by the frequency and duration of nucleic acid collection, the intensity of nucleic acid collection is relatively high and overload is more common. Among them, 79.1% were overloaded. Previous research has shown that prolonged and intensive work tends to worsen medical personnel's mental health (51). In addition, the heavy workload may make medical staff more susceptible to illness and the shifts in the outside environment will have an impact on collectors' attitudes. Previous studies have shown that the weather to some extent affects the psychological two important factors are the season and outdoor time (52). In January, the temperature outdoor Xi'an was relatively low, and the collecting staff worked longer in the low-temperature environment, which was more likely to lead to negative emotions than the more comfortable indoor environment.

## 5. Conclusion

The public was poorly informed about the mental health implications of the SARS pandemic when it first appeared in China, and those who need it did not receive any specific psychological support. In public health emergencies, the intensity and duration of medical staff increases, the risk of infection increases, and they face greater physical strength and mental stress, which leads to the occurrence of depression and anxiety, which is seriously affected sleep quality (53, 54). Staff members who performing nucleic acid collection always get physically and mentally exhausted during a protracted period of epidemic preparation, particularly during line-points closed-loop management. The suggestions are as follows. First, the staff can engage in appropriate physical exercise during closed-loop management to reduce psychological stress and relieve psychological tension. Second, relevant management departments should strengthen human resources to improve detection efficiency, optimize nucleic acid collection process, reduce the workload of nucleic acid collection staff, and improve the working environment for nucleic acid collection. Finally, the mental health status of nucleic acid collection medical staff should be investigated regularly and corresponding psychological assistance measures should be taken to alleviate depression, anxiety and sleep disorders of nucleic acid collection personnel under closed-loop management.

## 6. Limitations

There are some limitations in this study. First, we used a practical sample strategy based on an online questionnaire survey, which made our sampling dependent on the online network environment and potentially prone to selection bias. Second, it has a cross-sectional design which limits the ability to interpret the causal relationships between the different variables in this study. The association and causality can be more accurately determined in randomized prospective research. Finally, since the scope of our study was restricted to Shaanxi Province and the sample size was modest, a bigger sample size is required for the validation of our findings.

## Data availability statement

The original contributions presented in the study are included in the article/supplementary material, further inquiries can be directed to the corresponding author.

## Ethics statement

The Ethics Committee of the Affiliated Hospital of Shaanxi University of Traditional Chinese Medicine, Shaanxi Province, China approved this study (Ethical number: 2022-0516). The patients/participants provided their written informed consent to participate in this study.

## Author contributions

MS implemented this study and was responsible for data collection and analysis and writing. XL, JY, and YK took part in the process of data collection. XH and ZL provided assistance in reviewing the manuscript. All authors contributed to the article and approved the submitted version.
